# Dynamic trends, spatial clustering, and multi-model projections of the global burden of Alzheimer’s disease and other dementias: an analysis of GBD 1990–2021 data to 2050

**DOI:** 10.3389/fnagi.2026.1661370

**Published:** 2026-01-30

**Authors:** Fangfang Xiang, Xiaorui Liu, Gang Chen

**Affiliations:** 1 Department of Anaesthesiology, Zhejiang University School of Medicine Sir Run Run Shaw Hospital, Hangzhou, China; 2 Department of Radiology, Zhejiang University School of Medicine Sir Run Run Shaw Hospital, Hangzhou, China

**Keywords:** aged (80 and over), Alzheimer’s disease and other dementias, estimated annual percentage change, global burden of disease, sex factors, socioeconomic factors, spatial clustering, time-series forecasting

## Abstract

**Background:**

Alzheimer’s disease and other dementias (ADOD) are a leading causes of disability and mortality among older adults worldwide. While the rising burden is recognized, comprehensive analyses of its dynamic growth rates, spatial clustering patterns, and comparative long-term forecasts remain limited, hindering targeted policy response.

**Methods:**

Using data from the Global Burden of Disease (GBD) 2021 study for individuals aged ≥60 years across 204 countries (1990–2021), we analyzed six burden indicators. We calculated age-standardized rates (ASR) and estimated annual percentage changes (EAPC) to quantify trends. Spatial clustering of EAPC patterns was performed using hierarchical clustering. Future burden to 2050 was projected using both exponential smoothing (ES) and autoregressive integrated moving average (ARIMA) models, with comparative analysis across sex, age, and Socio-demographic Index (SDI).

**Results:**

From 1990 to 2021, global ADOD burden increased markedly in absolute terms. EAPC analysis revealed accelerated annual growth (>1%) in East Asia and Eastern Europe, surpassing the global average. Spatial clustering identified four distinct geographic archetypes, with rapid-growth clusters spanning middle-income regions in Latin America and Southeast Asia. Women and adults aged ≥80 years, especially those ≥95, bore a disproportionately high and increasing burden. Both ES and ARIMA models projected a continued rise in absolute burden to 2050, forecasting a near-doubling of the disease burden (DALYs) among women.

**Conclusion:**

The global ADOD burden is escalating with pronounced dynamic heterogeneity in growth velocity and distinct spatial patterns. Our multi-model projections warn of a mounting crisis, disproportionately impacting women, the oldest-old, and rapidly aging middle-income regions. Public health strategies must evolve from static assessments to dynamic surveillance and geographically tailored interventions, with urgent investment in prevention and care systems in high-growth clusters.

## 1 Introduction

Alzheimer’s disease and other dementias (ADOD) comprise a group of neurodegenerative disorders characterized primarily by progressive cognitive decline. ADOD is one of the leading causes of functional impairment and dependence among the global elderly population (Hugo and Ganguli, 2014; Lane et al., 2018; Scheltens et al., 2021). Clinically, affected individuals commonly present with memory loss, language disturbances, disorientation, personality changes, and a gradual loss of capacity for activities of daily living (Ramachandran et al., 2021). As the disease advances, these symptoms progressively worsen, ultimately resulting in a complete loss of independence. The impact of ADOD extends far beyond the affected individuals, imposing substantial psychological and economic burdens on family caregivers and presenting persistent challenges to healthcare systems worldwide.

According to estimates by the World Health Organization (WHO), there are currently approximately 55 million individuals living with dementia globally, a figure projected to reach 78 million by 2030 and 139 million by 2050 (Zhang et al., 2024b; Shin, 2022). Dementia has now become the seventh leading cause of death globally, further underscoring its growing public health significance (Zhang et al., 2024b; Arthurton et al., 2025). Worldwide, approximately 5 to 10% of individuals aged 60 years and older are affected by dementia, with Alzheimer’s disease accounting for 60 to 80% of all cases (Subramaniam et al., 2015; Rostagno, 2022; Alzheimer's Association, 2024). Notably, over 60% of dementia cases occur in low- and middle-income countries (LMICs), highlighting significant global inequities in healthcare resource allocation and the particular vulnerability of developing nations in confronting the challenge of ADOD (Stephan et al., 2020).

Global population aging has accelerated ADOD prevalence trends. Since 1990, both the number of cases and deaths attributed to ADOD have increased substantially, with the total number of patients in 2021 representing a roughly 160% increase compared to 1990 and mortality rising by 115% over the same period (Murray, 2024). Beyond these overall figures, the epidemiology of ADOD exhibits marked heterogeneity across regions and subpopulations. Regional studies have highlighted substantial disparities; for instance, prevalence rates among adults aged ≥65 years in Western Europe (8.46%) are more than double those in Southeast Asia (4.04%), likely due to differences in aging trajectories and access to preventive care (World Health Organization, 2021). A particularly concerning trend is the growing prevalence of early-onset dementia (EOD, onset before age 65), whose incidence and prevalence have more than doubled over the past three decades (Murray, 2024). This form of dementia carries a disproportionate societal impact, as it strikes individuals in their prime working years, leading to significant productivity loss and disrupting families with dependent children (Hendriks et al., 2021). Clinically, it is often characterized by non-amnestic symptoms like executive dysfunction or behavioral changes, frequently leading to diagnostic delays and misdiagnosis (de Vugt and Koedam, 2023). Etiologically, EOD exhibits a stronger genetic component and a more diverse neuropathological profile than late-onset dementia, adding layers of complexity to its diagnosis and management (Bateman et al., 2023; Onyike and Diehl-Schmid, 2023). These factors collectively exacerbate disease progression and caregiver burden, underscoring the critical need for targeted research and support systems (Van Vliet et al., 2011). Beyond age-related shifts, ADOD epidemiology also exhibits marked variations by sex—with women showing higher prevalence but lower mortality—and across geographic regions (Li et al., 2022; Xu et al., 2025a).

Forecasting the future burden of ADOD is critical for public health planning. Although existing models (e.g., cohort-based projections, machine learning approaches) have provided valuable insights, they possess notable limitations. Cohort models struggle to account for rapid demographic changes, while data-intensive machine learning methods are frequently constrained by the sparse and inconsistent data availability in many LMICs (Salcher-Konrad et al., 2023). Moreover, many existing global forecasts rely on single modeling approaches, which may not fully capture the uncertainty and variability in long-term trends.

While the Global Burden of Disease (GBD) Study has provided extensive epidemiological data on ADOD, and several studies have reported global or regional burden levels, critical gaps remain (GBD 2019 Dementia Collaborators, 2022; Zhang et al., 2024b; Guo et al., 2025). First, most existing research focuses on static estimates of burden rather than quantitative analysis of change rates—specifically, the Estimated Annual Percentage Change (EAPC)—and its dynamic variations across sex, age groups, Socio-Demographic Index (SDI), and regions. This limits the understanding of how burden disparities evolve over time. Second, systematic and comparative projections of future ADOD trends using robust time-series models [e.g., Exponential Smoothing (ES), Autoregressive Integrated Moving Average (ARIMA)] remain scarce, particularly for low-SDI regions. Third, analyses of geographic heterogeneity and spatial clustering of ADOD burden are insufficient, which is essential for identifying high-priority regions for intervention.

The work by Hao and Chen (2025) has provided a valuable longitudinal perspective on ADOD burden through 2021, employing joinpoint regression to identify significant trend transitions. To build upon this and address remaining limitations, our study introduces a complementary analytical framework focused on three underexplored dimensions essential for intervention targeting: first, a shift from describing when trends change to quantifying their annual growth velocity (EAPC) across fine-grained subgroups; second, the identification of spatial clusters based on these dynamic growth patterns, moving beyond static regional comparisons; and third, a comparative assessment of multiple time-series forecasting models (ES and ARIMA) to enhance the robustness of long-term projections.

To address these gaps, this study utilizes GBD 2021 data with three primary objectives: (1) to quantify temporal trends and EAPCs of ADOD burden across key demographics and regions; (2) to generate and compare robust forecasts of future burden using ES and ARIMA models; and (3) to identify geographic clustering patterns based on dynamic burden trajectories. By integrating trend analysis, comparative forecasting, and spatial assessment, this study aims to provide a nuanced evidence base for targeted interventions.

While landmark studies have provided crucial baseline estimates and explored specific aspects of ADOD, a comprehensive analysis integrating long-term dynamic trends (EAPC), comparative forecasting, and spatial clustering within the GBD 2021 framework remains lacking (Hendriks et al., 2021; GBD 2019 Dementia Collaborators, 2022). Our study builds upon this foundation by offering a more temporally refined (1990–2021), analytically layered (EAPC, dual-model forecasting, clustering), and spatially explicit investigation into the evolving global burden of ADOD.

## 2 Materials and methods

### 2.1 Data acquisition

All data in this study were sourced from the GBD 2021 database, encompassing core epidemiological indicators related to ADOD, including DALYs, YLLs, YLDs, prevalence, incidence, and mortality (Rudd et al., 2020). The study population comprised individuals aged 60 years and older worldwide, stratified into five-year age groups (60–64, 65–69, 70–74, 75–79, 80–84, 85–89, 90–94, and ≥95 years). Data analyses were further stratified by sex (male, female) and by region (global and 204 individual countries and territories) (Murray, 2022). All datasets were retrieved through the official GBD data interface (Global Health Data Exchange, GHDx), ensuring comprehensiveness and comparability.

### 2.2 Age-standardized rates and trend analysis

Age-standardized rates (ASR, per 100,000 population) utilized in this study were directly calculated based on GBD’s standardized methodology, employing the GBD global standard population to adjust for the influence of differing age structures and facilitate comparability across populations and regions (Jiang et al., 2023; Lv et al., 2024). The ASR was computed as follows:


$$\mathrm{ASR}=\frac{\sum (\mathrm{ai}\times \mathrm{wi})}{\sum \mathrm{wi}}$$

(where *ai* is the age-specific rate in group *i* and *wi* denotes the corresponding standard population.)

To assess temporal trends from 1990 to 2021 across countries/regions, SDI strata, sexes, and age groups, we applied a log-linear regression model to the ASR data to estimate the EAPC and corresponding 95% confidence intervals (CI) (Pu et al., 2023; Sun et al., 2024). The regression model is as follows:


ln(ASR)=α+β×year+ε

(where β is the regression coefficient, and ε is the random error term.)

The formulas for calculating EAPC and its 95% CI are as follows:


EPAC=100×[exp(β)−1]

95%CI=100×[exp(β±1.96×SE)−1]

(where SE denotes the standard error of β.)

A trend was defined as significantly increasing if the lower bound of the 95% CI was greater than zero, significantly decreasing if the upper bound was less than zero, and otherwise considered stable.

### 2.3 Clustering analysis and predictive modeling

EAPC values of ASR for all regions and countries were standardized using z-scores. Euclidean distance and hierarchical clustering (complete linkage) methods were used to classify regions or countries into four distinct categories. The optimal number of clusters was determined by inspecting the dendrogram for natural divisions and seeking a solution that provided clear epidemiological interpretation and regional coherence, with dendrograms constructed to illustrate geographic heterogeneity and similarity in disease burden trends (Hu and Yang, 2024).

For sex-stratified analyses, historical data from 1990 to 2021 were modeled using ES and ARIMA models to fit and forecast trends in ASR and case numbers. Projections were extended through 2050, and corresponding predicted values and 95% prediction intervals were reported (Wafa et al., 2024; Zhang et al., 2024a; Zhang et al., 2025).

For the ES model, the Holt’s damped trend variant was adopted, and the damping parameter (∅) was optimized via 5-fold cross-validation (ranging from 0.8 to 0.95), with a final value of 0.9. Its equations are as follows:


$$S_{t}=\alpha y_{t}+(1-\alpha )(S_{t-1}+\emptyset b_{t-1})$$

$$b_{t}=\beta (S_{t}-S_{t-1})+(1-\beta )\emptyset b_{t-1}$$

$${\hat{y}}_{t+h}=S_{t}+(\emptyset +\emptyset^{2}+\ldots \ldots +\emptyset^{h})b_{t}$$

(where $y_{t}$ denotes the observed value at time t, $S_{t}$ represents the smoothed estimate, $b_{t}\;$ is the trend component, α and β are the smoothing parameters, and ∅ (set at 0.9) is the damping factor governing the rate of decay for the trend over time. The parameter h represents the forecast horizon, indicating how many periods into the future the model is projecting.)

For the ARIMA model, the optimal order was selected using the Akaike Information Criterion (AIC), represented as ARIMA (*p*, *d*, *q*)—*p* = order of the autoregressive terms, *d* = number of differences required for stationarity, and *q* = order of the moving average terms, and its equations are as follows:


$$\emptyset (B){\left(1-B\right)}^{d}X_{t}=\varepsilon \theta {\left(B\right)}_{\varepsilon_{t}}$$

(where ∅(B) and θ (B) represent the autoregressive and moving average polynomials, respectively, B is the backshift operator, and $\varepsilon_{t}\;$ is the white noise error term.)

### 2.4 Statistical analysis

Normality of ASR and case numbers in different groups (sex, age, SDI, region, etc.) was evaluated using the Shapiro–Wilk test. For normally distributed continuous variables, one-way analysis of variance (ANOVA) was used for group comparisons; for non-normally distributed variables, the Kruskal-Wallis H test was applied. Associations between continuous variables were analyzed using Pearson’s correlation coefficient (for normal distributions) or Spearman’s rank correlation coefficient (for non-normal distributions), with correlation coefficients (*r*) and corresponding *p*-values reported. All estimates are presented with 95% CI, derived from the GBD database or calculated using the normal distribution of standard errors. Data processing and statistical analyses were conducted using R software (version 4.3.3) and relevant statistical and visualization packages. All statistical tests were two-sided, with *p* < 0.05 considered statistically significant.

## 3 Results

### 3.1 Determinants of the global burden of Alzheimer’s disease and other dementias

**Figure 1:**
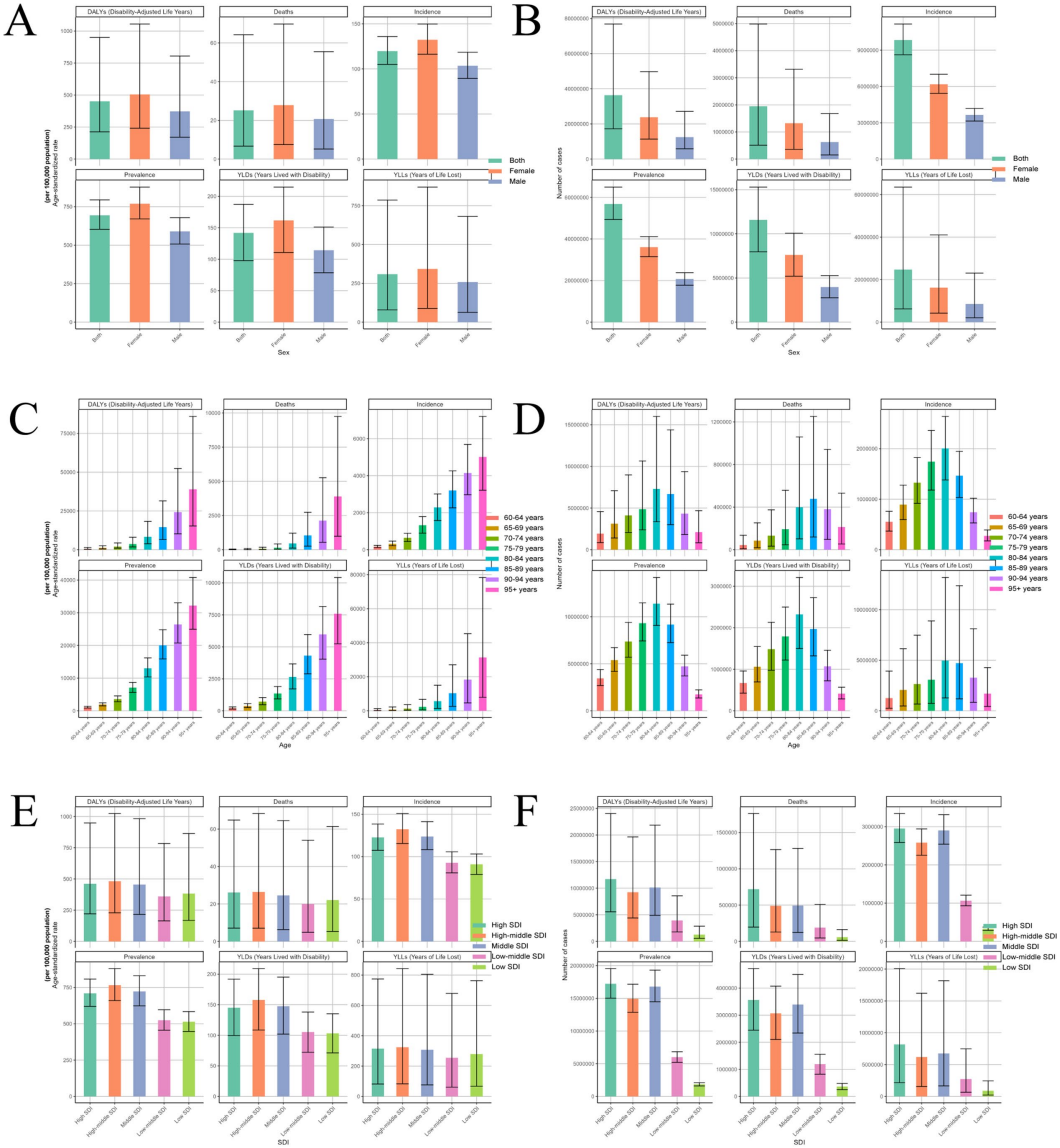
Determinants of the global burden of ADOD by sex, age, and SDI in 2021. This figure presents six core burden indicators of ADOD stratified by sex, age group, and SDI in 2021. **(A, C, E)** ASR (per 100,000 population) for each subgroup. **(B, D, F)** Absolute numbers of cases for each subgroup. **(A,B)** Stratified by sex. **(C,D)** Stratified by age group. **(E,F)** Stratified by SDI region. ADOD, Alzheimer’s disease and other dementias; SDI, Socio-demographic Index; ASR, age-standardized rate; DALYs, disability-adjusted life years; YLLs, years of life lost; YLDs, years lived with disability.

Analysis of the global burden of ADOD in 2021 reveals profound impacts of sex, age, SDI, and regional variation. Women exhibited substantially higher ASRs than men for prevalence and YLDs (Figure 1A). For example, women’s ASR of DALYs reached 504.87, markedly higher than men’s 372.53. In contrast, sex differences in mortality and YLLs ASRs were less pronounced. In absolute terms, women consistently bore a higher burden across all metrics (Figure 1B). In 2021, the number of DALYs among women was 23.81 million, nearly double that of men (12.52 million); similarly, deaths among women (1.33 million) exceeded those among men (0.63 million).

The burden of ADOD increased sharply with age, particularly among the oldest-old (≥95 years), with all indicators rising substantially in this group (Figure 1C). The ASR of DALYs escalated from 602.52 (60–64 years) to 8,323.84 (80–84 years). Over the same age span, the absolute number of cases rose from approximately 1.93 million to 7.29 million. Notably, the 80–84 and 85–89 age groups experienced the highest burden (Figure 1D).

With regard to SDI, both absolute burden and ASR increased in parallel with SDI levels (Figures 1E,F). High-SDI countries demonstrated the highest ASR for prevalence (709.47), with 17.22 million prevalent cases, followed by high-middle SDI countries (ASR: 766.20). For mortality, ASRs were 26.42 (high-middle SDI) and 26.21 (high SDI)—both significantly higher than low-SDI countries (22.07). The YLD ASR in high-middle SDI countries was 157.86, considerably greater than in low-SDI settings (103.45). YLL ASRs were 315.60 (high SDI) and 323.85 (high-middle SDI) versus 279.59 (low-SDI). Although low-SDI countries reported relatively lower absolute numbers (e.g., 58,600 deaths and 328,700 incident cases), their ASRs remain noteworthy, especially in the context of mortality and YLLs.

Regionally, developed areas with advanced health systems (e.g., Europe and North America) tended to exhibit higher burden across all indicators (Supplementary Figure 1). For instance, the “High-Performing Health System” region reported 3.94 million incident cases (ASR: 120.67), 933,700 deaths (ASR: 25.55), 22.82 million prevalent cases (ASR: 694.91), 4.73 million YLDs (ASR: 142.47), and 10.69 million YLLs (ASR: 307.88). In contrast, Africa, while exhibiting a comparable ASR for prevalence (589.98) and YLLs (303.90), had substantially lower absolute case numbers (2.74 million prevalent cases, 1.25 million YLLs).

### 3.2 Geographic patterns in the global burden of Alzheimer’s disease and other dementias

**Figure 2:**
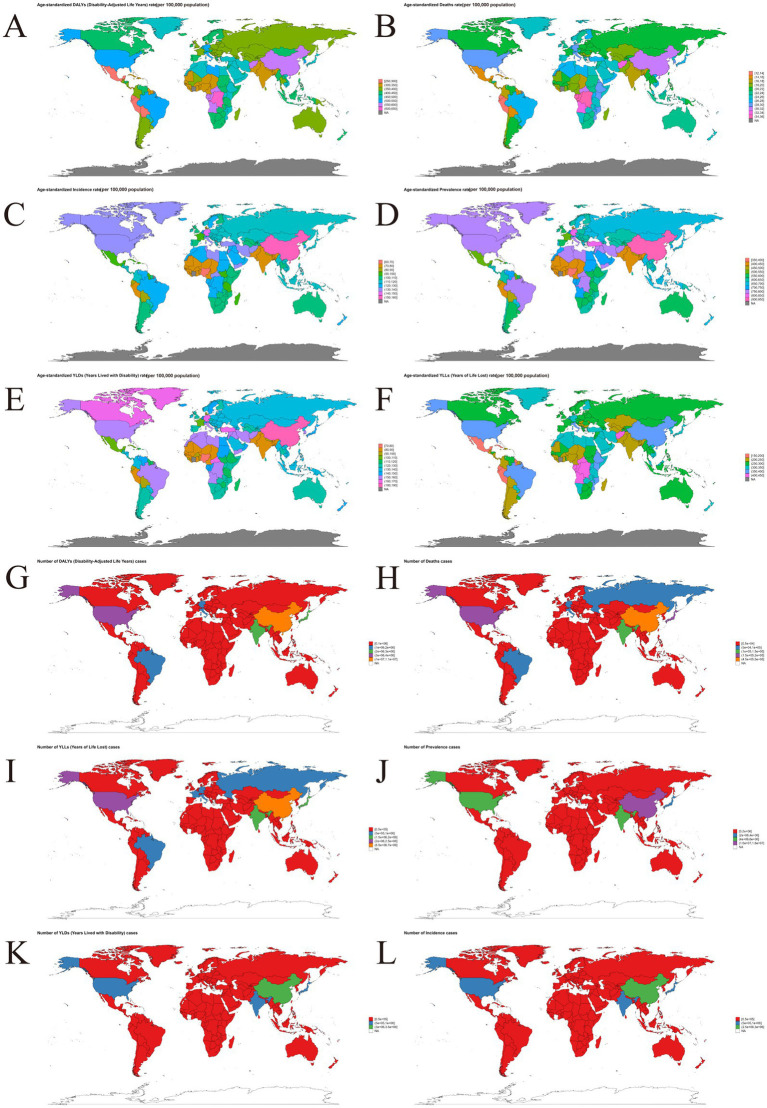
Geographic patterns in the global burden of ADOD in 2021. This figure presents the ASRs and absolute numbers of ADOD burden by country in map form. **(A–F)** Global distribution of ASR (per 100,000 population) for the six core health indicators (deaths, incidence, prevalence, YLDs, YLLs, DALYs). **(G–L)** Global distribution of the absolute number of cases for the six core health indicators. ADOD, Alzheimer’s disease and other dementias; ASR, age-standardized rate; YLDs, years lived with disability; YLLs, years of life lost; DALYs, disability-adjusted life years.

The burden of ADOD showed pronounced spatial heterogeneity across countries (Figures 2A–L). Maps of ASR reveal that high-income nations and regions with advanced population aging consistently exhibit elevated ASRs, with North America, Western Europe, Australia, and selected Asian countries being particularly prominent (Figures 2A–F). For YLDs, ASRs exceeded 160 in countries such as China (185.63), Germany (168.76), and Turkey (167.23). Regarding prevalence, China (900.82) and Lebanon (828.25) recorded some of the highest ASRs globally. In contrast, certain Central African and Central Asian countries exhibited relatively high ASRs for mortality and YLLs. For example, the Democratic Republic of the Congo (DRC) reported a deaths ASR of 35.44 and a YLLs ASR of 445.74. For DALYs, DRC (600.24), Gabon (588.29), Afghanistan (577.72), and the Republic of the Congo (ROC) (572.08)—all low-income countries in Africa and Asia—ranked among the highest globally. When examining absolute case numbers (Figures 2G–L), populous nations emerged as global “hotspots” for ADOD burden. China led the world in prevalent cases (16.99 million), followed by the United States (US) (4.88 million) and India (4.17 million). For YLDs, China reported 3.46 million cases, with the US and India contributing 978,200 and 811,200 cases, respectively. In terms of mortality, China again ranked first with 491,800 deaths, followed by the US (198,000) and Japan (172,800).

### 3.3 Sex- and age-specific differences in the burden of Alzheimer’s disease and other dementias

**Figure 3:**
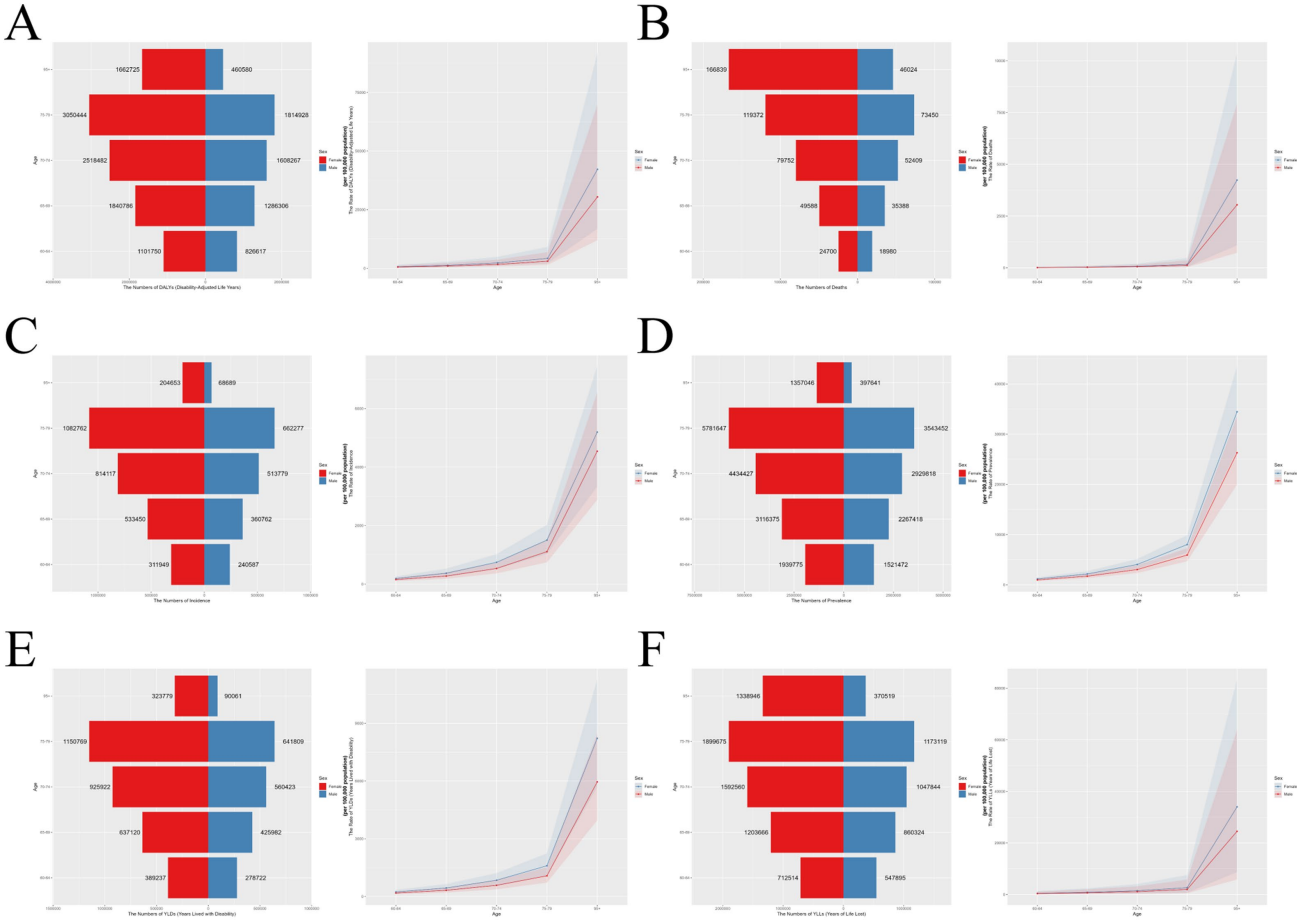
Sex- and age-specific differences in the global burden of ADOD in 2021. This figure details the burden of ADOD across six health indicators by sex and age group in 2021. **(A–F)** Each panel corresponds to one health indicator (prevalence, incidence, YLDs, YLLs, DALYs, deaths). Bar charts (left) show the absolute number of cases for males and females across age groups. Line graphs (right) show the trends in ASR (per 100,000 population) across age groups. ADOD, Alzheimer’s disease and other dementias; ASR, age-standardized rate; YLDs, years lived with disability; YLLs, years of life lost; DALYs, disability-adjusted life years.

This study systematically examined sex- and age-related disparities in six key health indicators of ADOD across 20 age groups in 2021, with particular focus on the elderly (60–64, 65–69, 70–74, 75–79, and ≥95 years; Figures 3A–F). Bar charts (left panels) illustrate the absolute numbers of cases by age group, while line graphs (right panels) display corresponding trends in ASR. Overall, all burden metrics—prevalence, incidence, YLDs, YLLs, DALYs, and deaths—increased sharply with advancing age, peaking among those aged 75 years and older. Across all age groups, women consistently bore a substantially higher burden than men in terms of case numbers, incidence, YLDs, YLLs, and mortality. These differences became especially pronounced in the oldest-old; for instance, among those aged ≥95 years, women experienced markedly higher mortality and overall disease burden compared to men. The right panel of the figure further demonstrates that, in every age group, women exhibited higher incidence rates, prevalence rates, YLD rates, YLL rates, and DALY rates than men.

### 3.4 Temporal trends and population heterogeneity (1990–2021)

**Figure 4:**
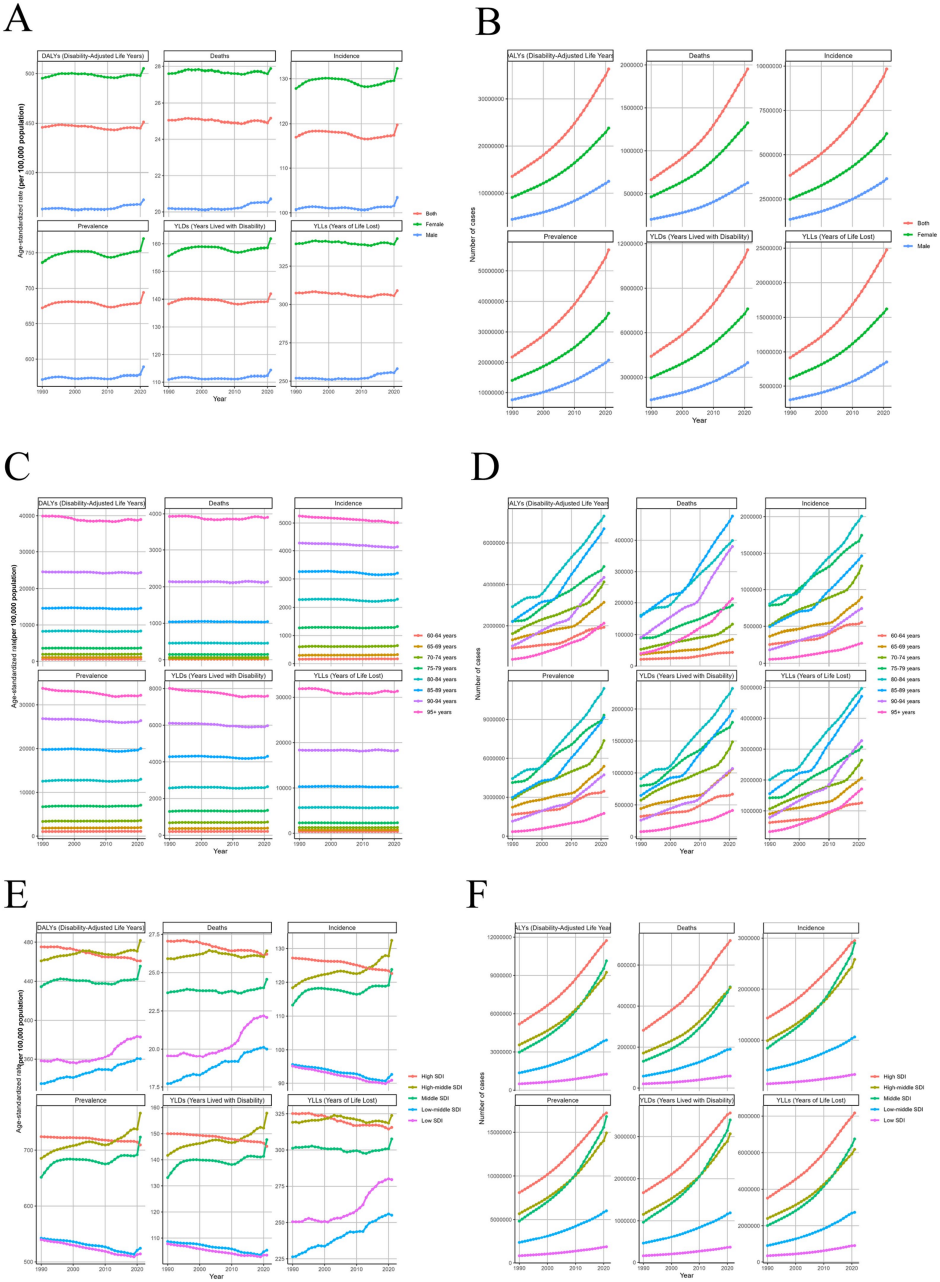
Temporal trends and population heterogeneity in the global burden of ADOD from 1990 to 2021. This figure presents the temporal trends in ADOD burden stratified by sex, age, and SDI from 1990 to 2021. **(A, C, E)** Trends in ASR (per 100,000 population) for each subgroup. **(B, D, F)** Trends in the absolute number of cases for each subgroup. **(A,B)** Stratified by sex. **(C,D)** Stratified by age group. **(E,F)** Stratified by SDI region. ADOD, Alzheimer’s disease and other dementias; SDI, Socio-demographic Index; ASR, age-standardized rate.

From 1990 to 2021, the global burden of ADOD exhibited a continuous and substantial increase, with significant upward trends in both ASR and absolute case numbers across all six major health metrics (Figures 4A–F). By sex, women consistently bore a greater burden than men in every indicator, with the most pronounced differences observed in DALYs and YLDs (Figures 4A,B). By age, the burden escalated markedly with advancing age, particularly among those aged 80 years and older. In the ≥95-year age group, the ASR for DALYs exceeded 40,000, with YLLs and mortality rates also reaching peak levels (Figures 4C,D). By SDI, countries with high and high-middle SDI levels generally had higher ASRs for incidence, prevalence, YLDs, and DALYs. In contrast, low-SDI countries exhibited rising trends in mortality-related indicators. Although the ASRs in low-SDI settings remained relatively low, the absolute number of cases has increased annually (Figures 4E,F).

### 3.5 Global EAPC-based clustering reveals four distinct regional patterns

**Figure 5:**
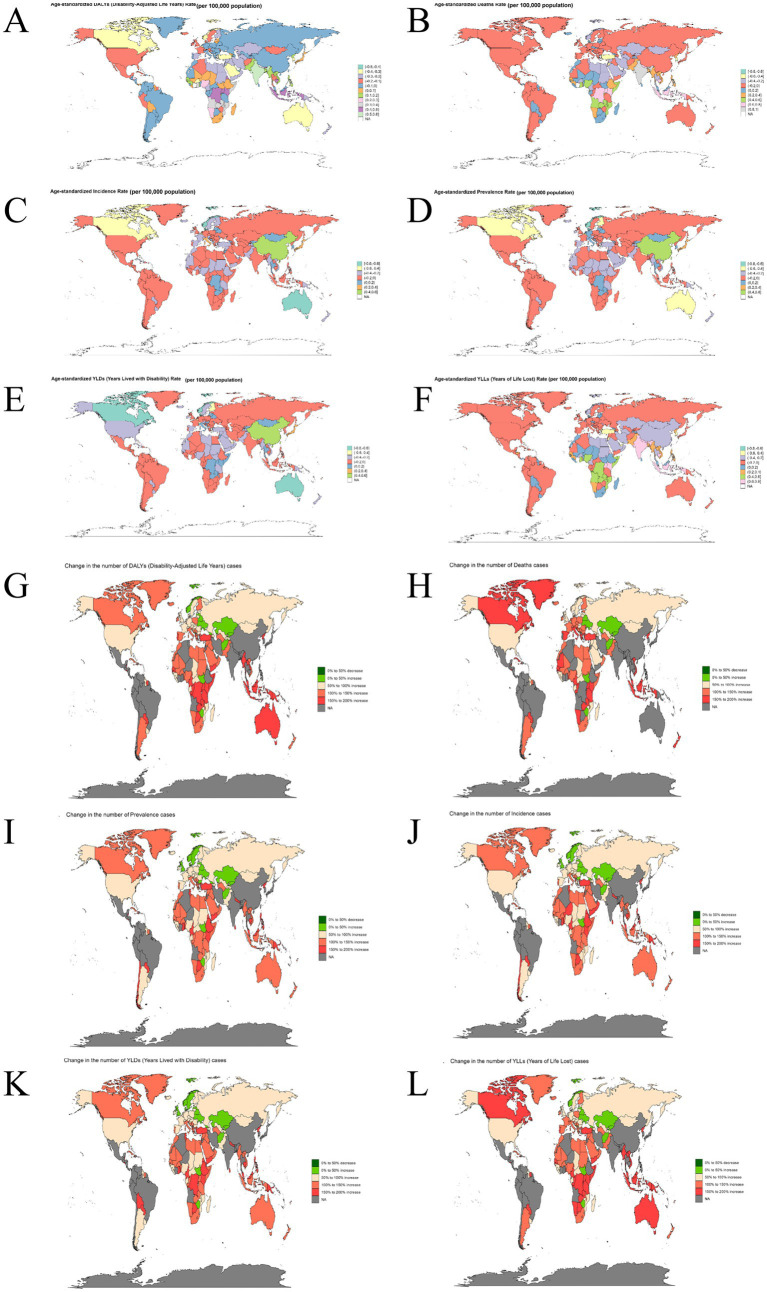
Global EAPC-based clustering reveals four distinct regional patterns of ADOD burden (1990–2019). This figure presents a clustering analysis based on the EAPC and spatiotemporal trends in ADOD burden change. **(A–F)** Global distribution maps of the EAPC (%) for the six health indicators from 1990 to 2019. **(G–L)** Global distribution maps of the percentage change (%) in the absolute number of cases for the six health indicators from 1990 to 2019. ADOD, Alzheimer’s disease and other dementias; EAPC, estimated annual percentage change.

Between 1990 and 2019, the global burden of ADOD exhibited complex spatiotemporal trends (Figures 5A–L; Supplementary Figure 2). Spatial clustering analysis based on the EAPC for six key health metrics of ADOD identified four major regional categories (Supplementary Figure 2). The first cluster (purple) comprised most developed regions (e.g., Europe, North America, upper-middle-income countries in Latin America) which shared similar EAPC patterns. The second cluster (green) encompassed Australia and parts of South Asia, displaying unique trajectories in disease burden. The third cluster (red) primarily included sub-Saharan Africa, the Middle East, and lower-middle-income regions in South Asia, where similar but less favorable trends prevailed. The fourth cluster (blue) aggregated Asia-Pacific regions (e.g., East Asia, high-income Asia-Pacific countries).

Analysis of dynamic trends using EAPC revealed that the majority of countries experienced sustained increases in all six burden indicators for ADOD, with striking geographic differences in the magnitude of change (Figures 5A–F). The most rapid growth rates were observed in Asia and Eastern Europe. For example, China reported EAPCs above the global average across multiple indicators—incidence (EAPC: 2.06, 95% CI: 1.53–2.58), prevalence (2.10, 1.55–2.65), YLDs (2.10, 1.53–2.68), YLLs (1.57, 0.89–2.27), DALYs (1.74, 1.09–2.40), and deaths (1.84, 0.93–2.76)—with most CIs not crossing zero. Similar patterns were seen in Japan (e.g., incidence: 2.06, 0.83–3.30; prevalence: 2.21, 0.96–3.46; YLLs: 2.15, 0.84–3.49), South Korea, Thailand, Taiwan, and several Eastern European nations (e.g., Albania, Armenia, Bosnia and Herzegovina, and Moldova), where EAPCs commonly exceeded 1. Additionally, several Latin American and Southeast Asian countries (e.g., Colombia, Brazil, Chile, Singapore, the Philippines, India, and Indonesia) showed positive EAPCs (often above 1) across various indicators (e.g., Colombia YLLs: 1.81 [1.13–2.48]; Singapore YLDs: 1.62 [1.10–2.14]). In contrast, the rate of increase in high-income countries was generally more modest. In US, EAPC point estimates for incidence (0.32, −0.29 to 0.93), prevalence (0.37, −0.25 to 0.98), YLDs (0.28, −0.33 to 0.90), YLLs (0.52, −0.13 to 1.18), DALYs (0.45, −0.19 to 1.09), and deaths (0.60, −0.10 to 1.30) were positive but most CIs crossed zero. European countries [e.g., France, the United Kingdom (UK), Germany, Sweden, the Netherlands] showed little variation, with many EAPCs close to zero. Some African and low-income countries (e.g., Afghanistan, Chad, Liberia, Nigeria, Mozambique, Madagascar) showed negative or non-significant EAPCs for certain indicators, with wide CIs suggesting slow growth or even declining trends (e.g., Afghanistan, incidence EAPC: −1.36 [−2.93 to 0.24]; deaths: −1.41 [−3.39 to 0.62]; YLLs: −1.51 [−3.25 to 0.26]).

Mapping trends in case numbers showed that nearly all countries experienced increases in absolute case counts from 1990 to 2019, particularly in Africa, Southeast Asia, and parts of the Americas and Oceania (Figures 5G–L). In most countries, the number of cases in all six indicators rose by 50 to 200%, with Australia, New Zealand, Canada, Chile, and many sub-Saharan African countries exceeding 150% increases. Some Nordic and Central/Eastern European countries (e.g., Finland and Sweden) reported only 0–50% increases in some indicators, while a few (e.g., Ukraine and Belarus) showed reductions of 0–50%. In China, most indicators increased by 50–100%.

Globally, most countries—especially those with low and middle incomes—are facing accelerating increases in disease burden. Notably, although ASR tended to stabilize or decline in most countries from 1990 to 2019, both EAPC and absolute case numbers indicate that the global burden of disease continues to rise.

### 3.6 Predictive analysis of health indicators based on ES and ARIMA models

**Figure 6:**
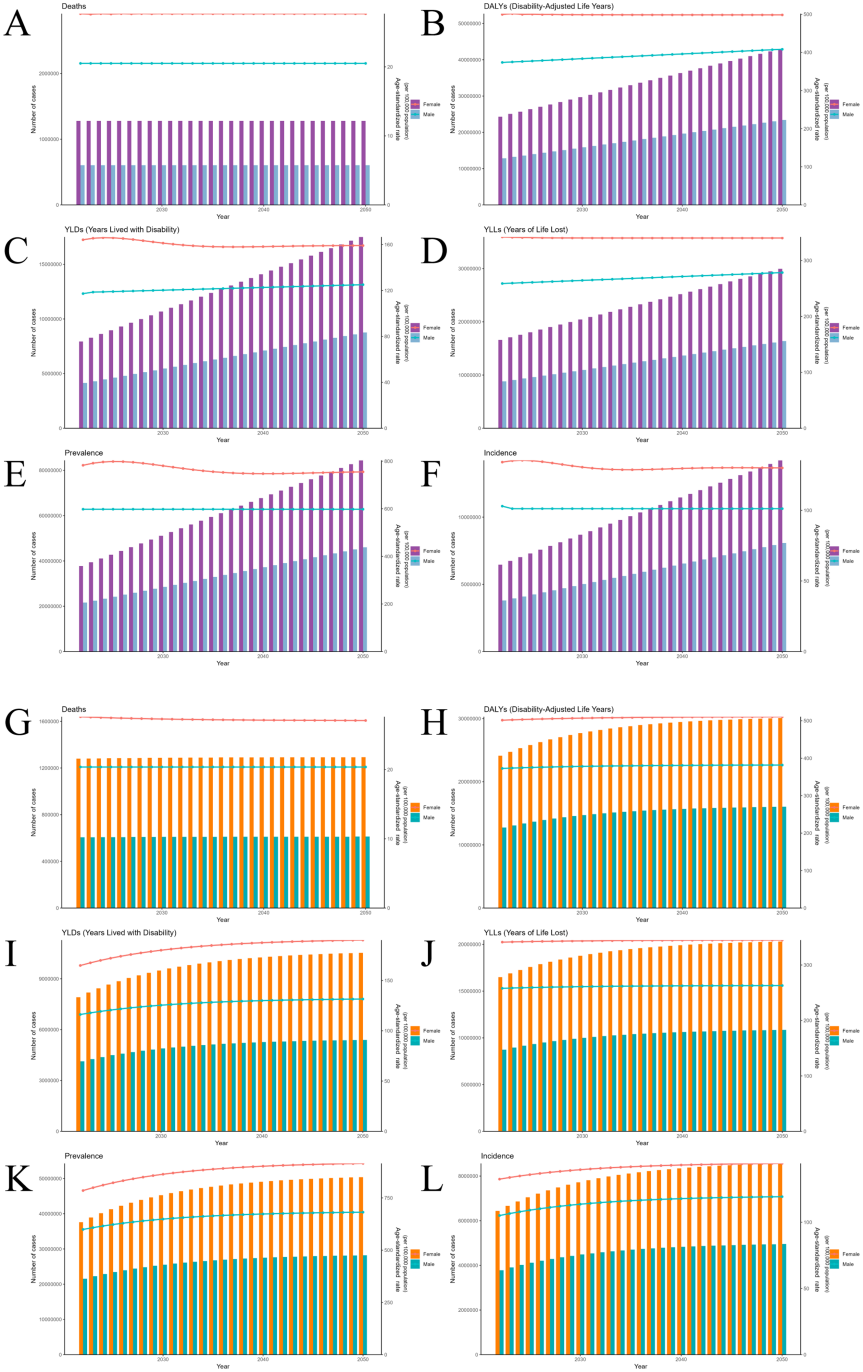
Predictive analysis of health indicators for ADOD based on ES and ARIMA models (2022–2050). This figure presents the projected trends in ADOD burden from 2022 to 2050 using the ARIMA and ES models. **(A–F)** Projections of the absolute number of cases for the six health indicators from the ARIMA model. **(G–L)** Projections of the absolute number of cases for the six health indicators from the ES model. The shaded areas represent the 95% prediction intervals. ADOD, Alzheimer’s disease and other dementias; ES, exponential smoothing; ARIMA, autoregressive integrated moving average.

Comparative projections from both ARIMA and ES models indicate that, over the next 29 years (2022–2050), the ASR of ADOD are expected to remain stable or show a slight decline across most indicators. However, against the backdrop of sustained global population aging and the rapid expansion of the oldest-old population, the absolute burden of these diseases is projected to increase substantially (Figures 6A–L).

The ARIMA model predicts a continued rise in the total number of deaths, incident cases, prevalent cases, YLDs, YLLs, and DALYs, with female figures consistently surpassing those of males in every dimension. For example, by 2050, the number of new cases among women is expected to exceed 13 million, prevalent cases will surpass 90 million, and DALYs will exceed 42 million. While the absolute numbers among men are comparatively lower, they are also projected to increase steadily (Figures 6A–F). The ES model further corroborates this trend, forecasting a sustained increase in all six health indicators from 2022 to 2050. The magnitude of increase in prevalence, incidence, YLDs, and DALYs is expected to be significantly greater among women (Figures 6G–L).

Both models consistently suggest that most ASRs will remain stable or decline slowly—especially for mortality and incidence—the projected decreases in YLL and DALY ASRs are minimal, with virtually no improvement anticipated among women. Compared to the ES model, the ARIMA model exhibits greater sensitivity and predictive accuracy in capturing subtle changes in disease burden, thus providing a more nuanced depiction of future trends. In summary, both models point to a continuously increasing global burden of ADOD over the coming decades, which is driven by accelerating population aging.

## 4 Discussion

Our analysis of GBD 2024 data confirms the escalating global burden of ADOD, characterized by a critical divergence between stable or declining age-standardized rates in some regions and persistently rising absolute numbers of cases, deaths, and disability metrics—a pattern largely driven by population aging. This finding aligns with existing literature but masks significant underlying heterogeneity in the velocity and distribution of burden growth, which our subsequent EAPC and clustering analyses reveal.

Compared to recent comprehensive analyses that have delineated key transition points and demographic drivers of ADOD burden (notably Hao and Chen, 2025), our study makes distinct contributions by shifting the analytical focus toward the velocity of change, dynamic spatial patterns, and comparative forecasting robustness. Whereas previous work effectively mapped when significant trend changes occurred, our EAPC analysis provides a continuous metric of how fast burdens are evolving annually, pinpointing that regions like East Asia and Eastern Europe are experiencing accelerated growth (>1% per year) significantly above the global average—a finding that refines static comparisons from earlier reports (GBD 2019 Dementia Collaborators, 2022). Beyond describing burden by SDI, our spatial clustering of these EAPC patterns reveals novel geographic archetypes, identifying, for instance, that certain middle-income regions in Latin America and Southeast Asia share rapid-growth trajectories with parts of East Asia, suggesting common underlying pressures (e.g., rapid demographic transition) that transcend income levels alone—a nuance less apparent in studies focusing on static prevalence maps (Li et al., 2022). In contrast to projections often reliant on single models, our application of dual, comparative time-series models (ES and ARIMA) strengthens the evidentiary basis for long-term forecasts, with both models converging on a trajectory of sharply rising absolute burden and a projected doubling of the female burden by 2050. Finally, our analysis delivers enhanced granularity on population disparities, systematically quantifying the disproportionate burden borne by women and the exponential risk gradient at the highest ages (≥80, particularly ≥95 years). While our primary analysis focuses on age ≥60, the demographic trends we identify underscore the expanding population at risk for EOD, highlighting an urgent need for future research with age-disaggregated data (GBD 2021 Young-Onset Dementia Collaborators, 2024). Together, these layered analyses refine the global burden landscape, emphasizing where (high-growth clusters), among whom (women, the oldest-old, and a growing at-risk population for EOD), and with what future trajectory the need for targeted action is most urgent.

The rise in ADOD burden has been especially pronounced in Asia, Eastern Europe, and selected countries in Latin America and Southeast Asia, where EAPCs for most indicators exceeded 1, and CIs rarely crossed zero, indicating significant upward trends (Wang et al., 2023). Notably, China, Japan, South Korea, Thailand, Taiwan, as well as Eastern European countries such as Albania, Armenia, Bosnia and Herzegovina, and Moldova, demonstrated EAPCs across incidence, prevalence, YLDs, YLLs, DALYs, and mortality that surpassed the global average (Deuschl et al., 2020; GBD 2021 Japan Collaborators, 2025). This pattern aligns with and extends the findings of regional studies and not only reflects accelerated population aging and expanding oldest-old cohorts, but also aligns with regional studies noting improved disease diagnosis, surveillance, and public awareness in these nations—a factor less emphasized in previous global analyses (Li et al., 2022; Wang, 2025). However, our analysis adds a crucial layer by demonstrating that growth rates in these regions surpass the global average, suggesting a compounding effect of demographic shifts and improving diagnostic capture. Latin American and some Southeast Asian countries—including Colombia, Brazil, Chile, Singapore, the Philippines, India, and Indonesia—also showed persistent increases in multiple indicators (EAPC > 1), further underscoring the influence of both demographic aging and socioeconomic development. By contrast, high-income countries in Western Europe and North America, such as the US, France, the UK, Germany, Sweden, and the Netherlands, exhibited limited changes in EAPCs, with most CIs spanning zero, indicating slower increases in burden (Feigin et al., 2021). This may be attributable to earlier intervention, robust prevention and rehabilitation systems (e.g., cardiovascular health initiatives), and improvements in health education—consistent with reports of modifiable risk factor mitigation reducing dementia growth in these settings (Xu et al., 2025b).

It is noteworthy that some low-income and less developed countries (e.g., Afghanistan, Chad, Liberia, Nigeria, Mozambique, Madagascar) exhibited negative or near-zero EAPCs for certain indicators, with wide CIs, indicating slow or even declining burden. Possible explanations include slower population aging, limited early diagnosis, insufficient public health investment, and factors such as population structure and migration. However, prior research has highlighted that actual burden in these countries may be underestimated due to shorter life expectancy and lower data quality—a limitation that must be considered when interpreting these trends, as our future projections suggest the burden will rise with upcoming demographic shifts (Xu et al., 2025b).

This study also identified significant sex and age heterogeneity. Across all health metrics, the burden in women exceeded that in men, with the greatest disparities observed in the oldest age groups (GBD 2016 Disease and Injury Incidence and Prevalence Collaborators, 2017; Fang et al., 2025). This extends findings from smaller cohort studies to a global scale, as women had higher absolute numbers and ASRs for DALYs, YLDs, and deaths—likely reflecting a confluence of biological (e.g., longer life expectancy, post-menopausal neuroprotective changes), social (e.g., caregiving-related stress), and possibly healthcare-seeking factors, rather than demographic structure alone (Mielke et al., 2023; Wang, 2025). The burden increased sharply in older age groups, especially those aged 80 and above, and was most pronounced among those 95 and older, highlighting the formidable challenges faced by “super-aged” societies (Agudelo-Botero et al., 2023; GBD 2021 Japan Collaborators, 2025; Zeng et al., 2025). In countries with high proportions of elderly, ADOD prevention and care have become central tasks for health and social security systems. Stratification by SDI indicated that high-SDI countries had higher ASRs and case numbers, driven by both the speed of aging and high diagnostic rates, while the rising burden in low-SDI countries—even with lower absolute numbers—emphasizes the global relevance of chronic disease burden across all development settings, supporting policy calls for LMIC-focused dementia care investment (Wang, 2025). Our findings are consistent with recent comprehensive burden analyses in the elderly, which identify ADOD as a leading contributor to the overall disease burden in the aging population worldwide (GBD 2021 Diseases and Injuries Collaborators, 2024).

Trends in case numbers and spatial distribution further demonstrated that, in recent decades, nearly all countries have experienced sustained increases in ADOD burden. In Africa, Southeast Asia, parts of the Americas, and Oceania, the number of cases for multiple indicators increased by more than 150%, representing a rapid escalation of health burden (Xu et al., 2025b). By contrast, increases in some Nordic and Central/Eastern European countries were more modest, or even negative for certain indicators, highlighting the influence of population structure, health system capacity, and prevention strategies on the evolution of burden. China showed a 50–100% increase in several metrics, reflecting its rapidly aging demographic and positioning ADOD as a leading chronic disease challenge in the decades ahead.

Our predictive models indicate that, barring major advances in intervention, both incidence and case numbers of global ADOD will continue to rise in the coming decades (Guo et al., 2025). Unlike some studies that used single models for projections, our dual ARIMA and ES models—which both demonstrating pronounced upward trends in deaths, incidence, prevalence, YLDs, YLLs, and DALYs—strengthen the reliability of these forecasts, with especially marked increases in women (Chen et al., 2025). Although some high-income countries have observed age-specific declines in incidence, likely related to improved education, better cardiovascular health, and lower smoking rates, the absolute number of older adults is rising rapidly worldwide. Our projections reinforce the warning that, by 2050, the global burden of ADOD will reach unprecedented levels (World Health Organization, 2021). This aligns with WHO forecasts but adds granularity on sex and regional differences, providing more targeted evidence for policy planning. Even if ASRs remain stable, the growing number of elderly will impose immense pressures on health systems.

This study has several limitations. First, while the GBD database employs systematic modeling (including DisMod-MR 2.1) to estimate epidemiological data across regions, the results may be affected by the availability and quality of primary data, leading to potential inaccuracies. Specifically, LMICs—which we identified as having growing but understudied ADOD burden—often have sparse surveillance data or incomplete case reporting. This may introduce greater uncertainty in estimates for these regions, particularly for EOD and YLDs, where clinical diagnosis rates are lower.

Second, this study adopts an ecological design focused on macro-level population trends, which limits our ability to disentangle the contributions of individual-level risk factors. Consequently, we cannot establish causal links between the observed burden disparities (e.g., by sex or age) and specific etiological factors. As highlighted in the introduction, factors such as genetic susceptibility, educational attainment, dietary patterns, and environmental exposures are known to influence ADOD risk, but our analysis cannot link these variables to the observed burden disparities (e.g., sex- or age-related differences in incidence). This inherent limitation of ecological studies precludes the inference of individual risk from our aggregate data.

Finally, our forecasting models (ES and ARIMA) rely on extrapolation from historical epidemiological trends (1990–2021). They do not account for potential disruptive events that could alter ADOD trajectories, such as breakthrough disease-modifying therapies, large-scale public health interventions (e.g., targeted dementia prevention programs), or major societal crises (e.g., pandemics, economic collapses) that may impact healthcare access or risk factor exposure. Additionally, the models do not incorporate future changes in population structure beyond aging (e.g., shifts in socioeconomic status or urbanization) that could further modify burden trends.

In conclusion, our comprehensive analysis of the global ADOD burden from 1990 to 2021, with projections to 2050, reveals three critical and actionable insights. First, the epicenter of the ADOD pandemic is shifting, with LMICs experiencing the most rapid growth in absolute case numbers, despite lower ASRs. Second, profound and persistent inequalities exist, with women and the oldest-old (≥80 years) carrying a disproportionate share of the burden. Third, distinct regional clustering of burden trends demands tailored public health responses.

Our findings imply that a one-size-fits-all global strategy is inadequate. High-income countries must strengthen long-term care systems and research into sex-specific risk factors. For LMICs, where the burden is rising most sharply, there is an urgent need to invest in building basic dementia surveillance, diagnosis capacity, and community-based care. This is particularly critical given that health systems in many LMICs are currently underprepared for the challenges of population aging, including the rising tide of dementia (Sheeh et al., 2024). Future research should leverage individual-level cohort data to disentangle the complex interplay of genetic, lifestyle, and social determinants of ADOD, and evaluate the cost-effectiveness of targeted interventions in high-risk populations and regions.

## Data Availability

Publicly available datasets were analyzed in this study. This data can be found at: Global Health Data Exchange, GHDx.
