# Supplementary material for: Dynamic trends, spatial clustering, and multi-model projections of the global burden of Alzheimer’s disease and other dementias: an analysis of GBD 1990–2021 data to 2050
Source: Front Aging Neurosci. 2026 Jan 30;18:1661370. doi: 10.3389/fnagi.2026.1661370 (PMC12901325; doi:10.3389/fnagi.2026.1661370)

**Supplementary Figure 1. This figure presents the ASRs and absolute numbers of ADOD burden by 21 GBD regions.** (A) ASR (per 100,000 population) for the six core health indicators (Deaths, Incidence, Prevalence, YLDs, YLLs, DALYs). (B) The absolute number of cases for the six core health indicators. ASR, age-standardized rate; ADOD, Alzheimer's disease and other dementias; YLDs, years lived with disability; YLLs, years of life lost; DALYs, disability-adjusted life years.


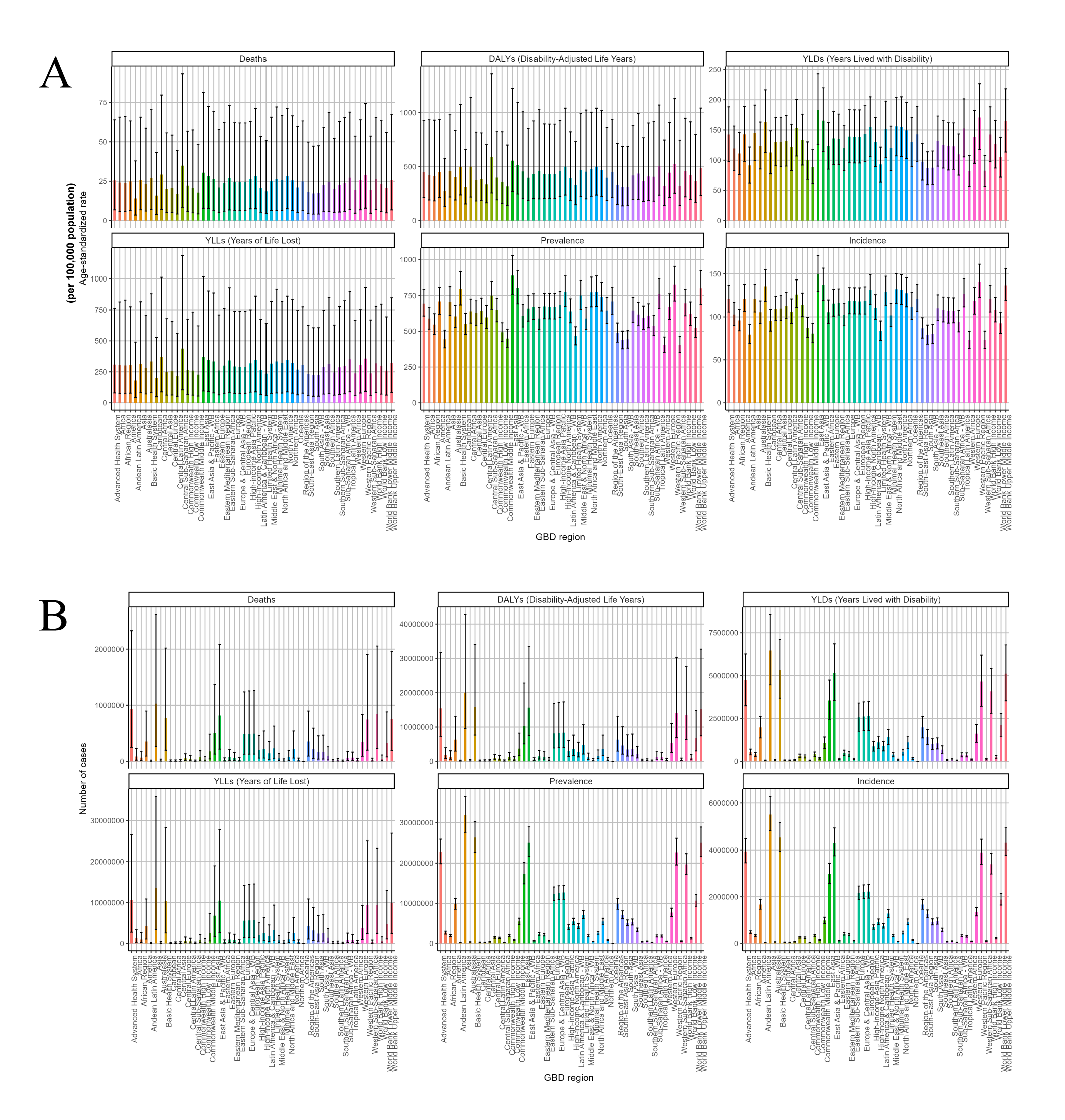


**Supplementary Figure 2. Hierarchical clustering dendrogram based on estimated annual percentage change (EAPC) values for the six health indicators, categorizing regions into four clusters.**


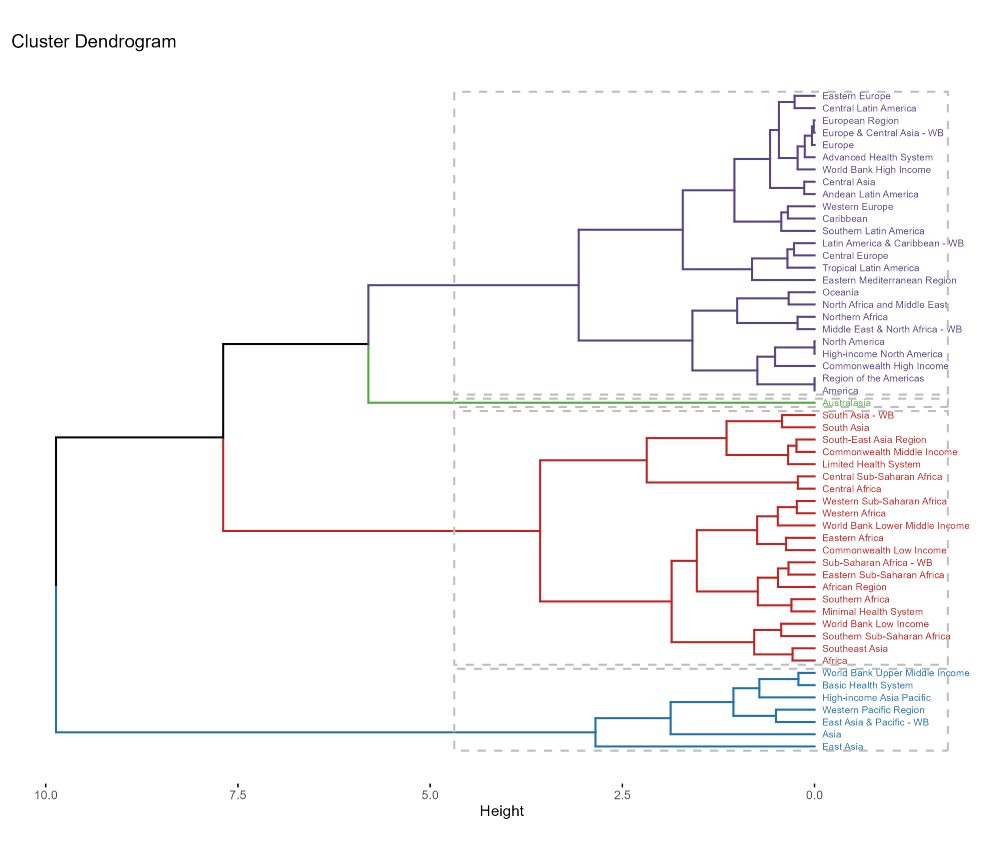

Supplement: Supplementary file 1 [file Table_1.DOCX]
